# The degree of prevalence of similarity between outer tropical cyclone rainbands and squall lines

**DOI:** 10.1038/s41598-018-26553-8

**Published:** 2018-05-29

**Authors:** Cheng-Ku Yu, Che-Yu Lin, Lin-Wen Cheng, Jhang-Shuo Luo, Chun-Chieh Wu, Ying Chen

**Affiliations:** Department of Atmospheric Sciences, National Taiwan University, Taipei, Taiwan

**Keywords:** Natural hazards, Atmospheric dynamics

## Abstract

Tropical cyclone rainbands (TCRs) are not only one of the most striking and persistent features of tropical cyclones (TCs) but also one of the major causes for extreme floods as TCs approach or encounter the land area. TCRs have been traditionally considered as manifestations of atmospheric waves initiated near the eyewall or close to the TC center. However, the limited evidence from recent studies showed the possibility for TCRs to develop squall-line-like characteristics in the outer region of TCs. In this study, the degree of the prevalence for this similarity is explored by radar and surface observations from a large set of 50 outer TCRs associated with 22 TCs as they approached Taiwan. The results indicate that around 58% of outer TCRs are similar to squall lines. These outer TCRs are generally characterized by convective precipitation, an obvious convergence zone between the band-relative rear-to-front flow and front-to-rear flow at low levels, either frontward or rearward tilting updrafts, and a surface cold pool signature. The frequent similarity between the outer TCRs and squall lines documented provides important insights into the formation of organized, heavy precipitation associated with TCs.

## Introduction

In addition to the eye and eyewall, tropical cyclone rainbands (TCRs) are the most striking and persistent feature of tropical cyclones (TCs)^1–8^. Outside the eyewall region, TCRs are a primary, concentrated region of the heaviest precipitation within TCs and play prominent roles in influencing the intensification or weakening of the storms^9–16^. TCR landfalls and the interactions between the topography and the TCs as they approach and move over landmasses are major causes of heavy rainfalls and/or severe floods in many different geographical locations^17–25^.

In the past few decades, significant efforts have been made by a large number of tropical cyclone studies to identify the possible origins of TCRs. The most well-known concept in both observational and theoretical studies is that the appearance of TCRs is considered to be a manifestation of atmospheric waves initiated near the eyewall or close to the TC center^26–38^. Despite the diversity of wave types proposed previously, these wave arguments, to a certain extent, have reasonably explained the formation, spiral nature, and propagation characteristics of TCRs. Having said so, different interpretations of TCR origins and propagation continue to emerge in the literature^5,39–43^, and a consensus has not yet been reached.

In contrast to TCR wave theories, there is still a vast gap in our knowledge regarding the physical processes conducive to the triggering and maintenance of the moist convection associated with TCRs. This is mostly due to the incomplete documentation of the complexity of convective processes inside the TC environment. One of the intriguing questions is whether the convective initiation and development of TCRs are similar to those of ordinary convective rainbands such as squall lines. Squall lines, according to the AMS Glossary of Meteorology, are usually referred to as a line of active deep moist convection and are a frequent and the most well-documented mode of precipitation organization within mesoscale convective systems^44^. The presence of pronounced cold pools at low levels is one of the most striking thermodynamic signatures associated with squall lines and many organized convective systems and represent a crucial convective forcing in the initiation and development of moist convection^45–48^.

Considerable aircraft observations have revealed some unique mesoscale structures of TCRs, which are tied to the inner-core vortex dynamics and are essentially different from those of squall lines^4,11,12,49–57^. The temperature deficit produced by precipitation evaporation within these documented TCRs was typically weaker than that of ordinary convective systems^11,12,48^. However, most of these previous case studies of TCRs dealt with the so-called “principal band”, a well-known rainband type that is typically located near the boundary between the inner core and outer region in TCs^5,7,8^. It is clear that these aircraft investigations are largely confined to the inner-core vicinity and thus provide a rather incomplete depiction of the entire spectrum of TCRs.

Because the convective behavior within TCs is fundamentally determined by the degree to which they are influenced by the inner-core vortex, TCRs may be conceptually classified into inner and outer rainbands. Theoretically, the inner TCRs are recently recognized to be more probably related to vortex Rossby waves^32,37^. However, the outer TCRs are traditionally considered as inertia-gravity waves^27–29,58^, although the outward propagation of the observed outer TCRs (several meters per second) seems much slower than the typical outward speed of inertia-gravity waves (several tens of meters per second)^38,42^. The frequent occurrence of inertia-gravity waves within TCs has also been reported in some recent published works^59,60^. The gravity-wave-like disturbances can propagate outward into the outer region of TCs, in distinct contrast to the vortex Rossby waves that exhibit the stagnation radius [~3 times the radius of the maximum wind (RMW)] in their radial propagation. However, the degree to which the outer TCRs resemble the inertia-gravity waves remains largely unclarified due to the lack of detailed observations^38^. The moist convection of the inner TCRs is strongly constrained by the inner-core vortex, generally moves with ambient rotational flow, and is rapidly filamented^3,61^. In contrast, the precipitation of the outer TCRs usually exhibit higher asymmetry as opposed to the quasi-circular geometry of the inner TCRs^3,5,8^. In addition, the outer areas of TCs usually possess larger convective available potential energy (CAPE) and lower humidity than the inner-core environment^62–66^, both of which would facilitate intense convection, the effect of precipitation evaporation, and the occurrence of potentially threatening and severe weather conditions. Consistent with this point of view, limited research evidence from studies of TCs indicates the likelihood for outer TCRs to develop squall-line-like airflow structures and a low-level cold-pool signature^66–72^. However, the degree of the prevalence for the appealing similarity between outer TCRs and squall lines has not been identified at this stage because only very few outer TCRs have been thoroughly studied and reported in the literature.

The primary objective of this study is to use long-term radar observations (2003~2015) to investigate a considerable number of outer TCRs from different TCs as they approach Taiwan, in an attempt to advance our general understanding of the outer TCRs and their possible similarities to squall lines. Taiwan is a well-known target for TCs originating over the northwestern Pacific Ocean and has established an excellent whole-island Doppler radar observing network in the 2000s. Both the above aspects make this geographical location unique for the investigation of the outer TCRs. The particular focus of the present study is to perform dual-Doppler radar analyses for a comprehensive TCR dataset to understand to what extent the squall-line-like convective structures are prevalent in the outer regions of TCs.

## Data, Methodology and Rainbands

### Radar measurements

**Figure 1:**
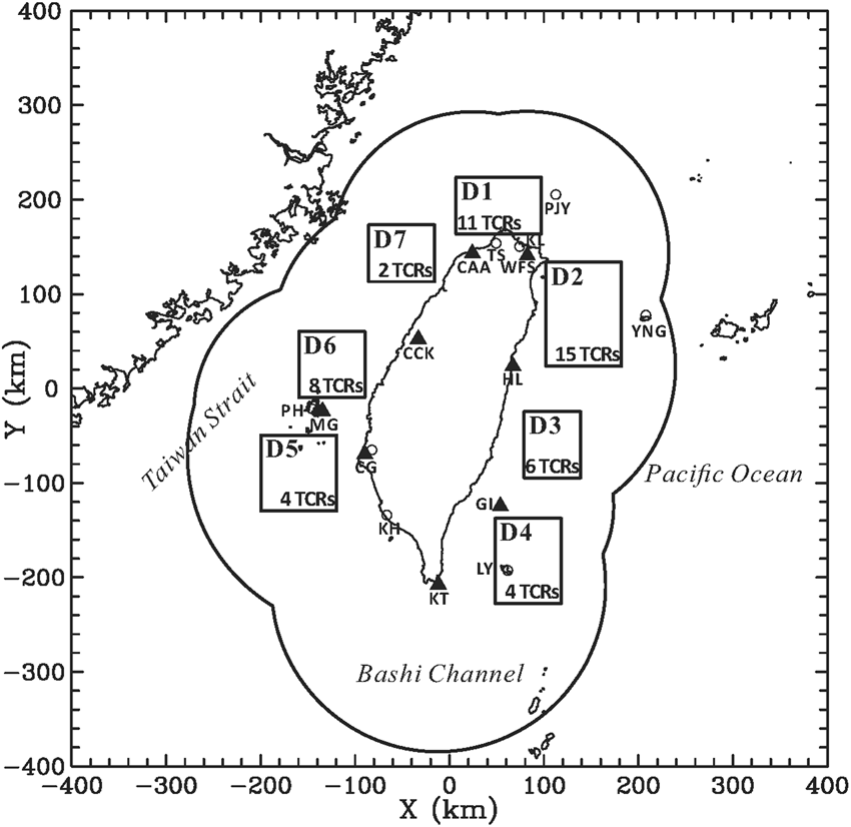
Radar and surface observations used in this study. The locations of the Doppler radars at Wu-Fen-San (WFS), Hua-Lien (HL), Chi-Gu (CG), Ken-Ting (KT), Civil Aeronautics Administration (CAA), Green Island (GI), Ma-Gong (MG), and Ching-Chuan-Kang (CCK) are denoted by triangles. The locations of the selected surface and island stations are denoted by the hollow circles. The seven inset boxes (D1~D7) surrounding the Taiwan Island indicate the dual-Doppler synthesis domains. The number of the outer TCRs available for dual-Doppler analysis within each of the synthesis domains is also indicated. The outer curve denotes the approximate data coverage for these radars. This map is generated by the NCAR Command Language (Version 6.4.0) [Software]. (2017). Boulder, Colorado: UCAR/NCAR/CISL/TDD. 10.5065/D6WD3XH5.

The primary datasets used in this study were provided by eight ground-based Doppler radars (see the locations in Fig. 1) available in Taiwan, including four S-band (10 cm) operational Doppler radars of the Central Weather Bureau (CWB) at Wu-Fen-San (WFS), Chi-Gu (CG), Ken-Ting (KT), and Hua-Lien (HL); one C-band (5 cm) operational Doppler radar of the Civil Aeronautics Administration (CAA) at Taoyuan International Airport; and three C-band (5 cm) operational Doppler radars of the Weather Wing of the Chinese Air Force at Green Island (GI), Ma-Gong (MG), and Ching-Chuan-Kang (CCK). The basic characteristics of these Doppler radars are indicated in Table 1. These radars provide volume scans of reflectivities and radial velocities with a temporal interval ranging from 5 to 30 min between each volume. The radars typically have 9–15 scanning elevations from 0.4° to 19.5°, with smaller elevation intervals roughly comparable to the beam width (~1°) at lower elevations. For a given radar, the maximum observational ranges may vary (Table 1) due to different scanning strategies for operational purposes during the long-term period of the study (2003–2015). The data coverage of these radars encompasses extensive regions, from Taiwan landmass to approximately 150–200 km offshore (cf. Fig. 1).

**Table 1:** Radar characteristics of the eight ground-based Doppler radars available in Taiwan.

Parameter	WFS	HL	CG	KT	CAA	GI	MG	CCK
Longitude (°E)	121.7728	121.62	120.0691	120.8469	121.2	121.4836	119.6262	120.6261
Latitude (°N)	25.0729	23.99	23.1477	21.9026	25.07	22.6525	23.5646	24.2526
Altitude (m)	766	63	38	41	27	284	48	203
Wavelength (cm)	10	10	10	10	5.31	5.33	5.3	5.33
Beamwidth (°)	0.95	0.95	0.95	0.95	0.857	1.02	0.93	0.95
Pulse repetition frequency (Hz)	857~1204	800~1300 (625,468)	800	1000~1200 (625,468)	1000,750	1180 (1180,885)	(1000,750) (1250,937)	(1000,750) (937,749)
Max Unambiguous velocity (m s^−1^)	22.0~31.0	21.2~50.0	21.2	26.4~49.5	39.8	15.6~46.8	39.7~49.6	37.5~40.0
Max range (km)	125~176	115~230	188	125~230	150	120	120~150	150~160
Along-beam range resolution (km)	0.25/1	0.25	0.25	0.25	0.25	0.5	0.5	0.5

For several radars, two pulse repetition frequencies (PRF) indicated in the brackets represent the adoption of dual PRF technique for extension of unambiguous velocities.

### Rainband identification

To identify the TCRs approaching Taiwan, a very large set of low-level plan position indicator (PPI) scans of the reflectivity are screened subjectively. The set of data is obtained from the available radars for all TCs during 2003~2015, for each of which CWB had issued warning reports. The identification of an outer TCR is based on the following criteria. First, an organized precipitation feature with a stronger (weaker) reflectivity intensity inside (outside) the band and with a length much greater than its width must be visually identified. Second, the reflectivity values along the bands should be greater than 20 dBZ and the boundary of the bands must be well determined via the horizontal reflectivity patterns. With these two criteria, the precipitation features such as relatively transient showers or light stratiform precipitation usually present within TCs could be effectively avoided. Third, the rainband segment should move well into the dual-Doppler synthesis domains (cf. Fig. 1) from the open ocean. To simplify the problem and mitigate topographic effects, those rainband segments that passed over the Taiwan landmass, either completely or partially, before moving into the synthesis domains are excluded. Fourth, the radial distances of the rainbands should be generally located beyond a threshold of 3 times RMW (provided by Joint Typhoon Warning Center) from the TC center. From dynamical perspectives, moist convection within ~3 times RMW is expected to be strongly influenced by inner-core rotational flow under strong filamentation effects^61,73^.

**Figure 2:**
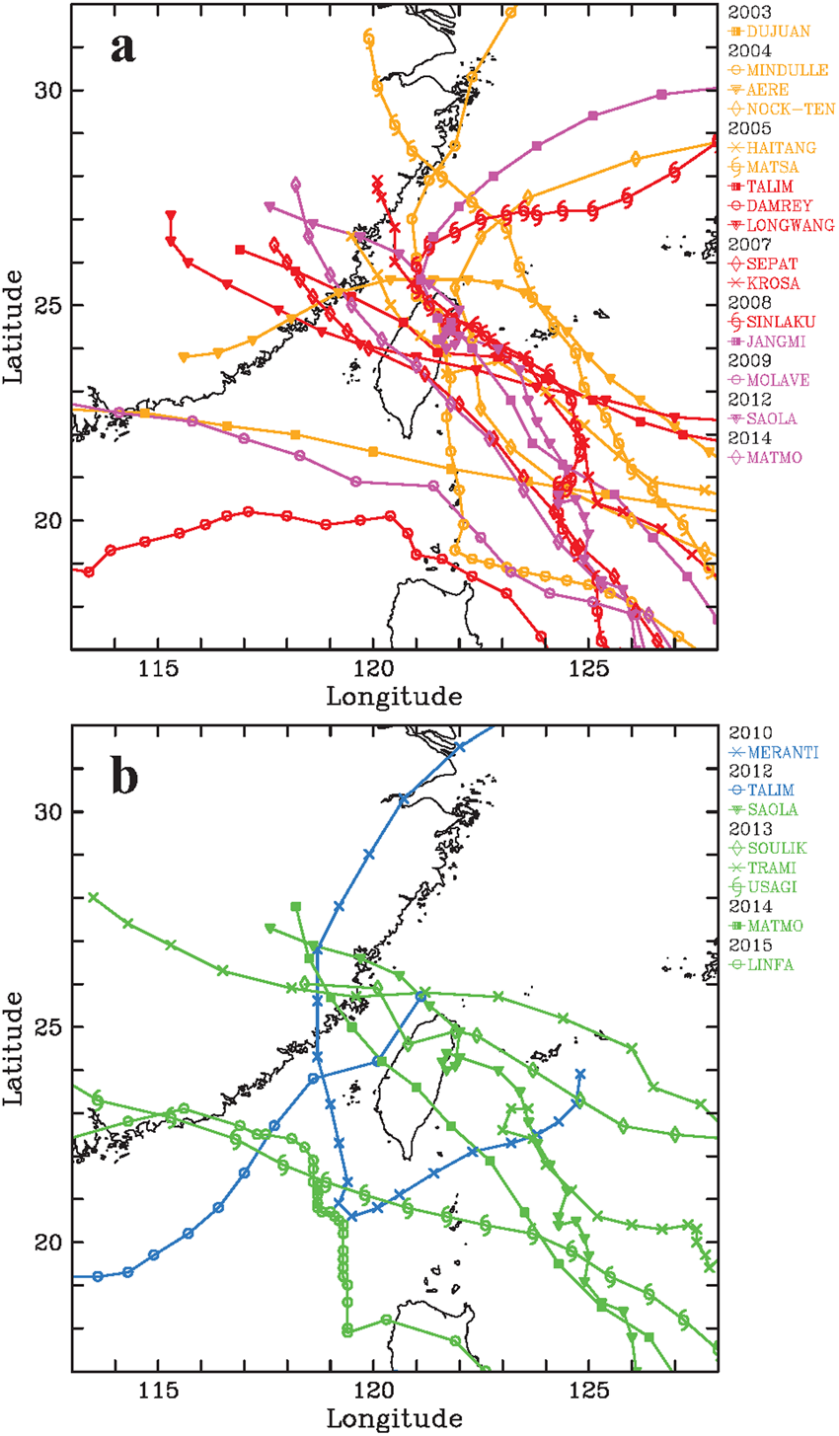
Best track of TCs associated with the outer TCRs identified in this study passing over the synthesis domains (**a**) D1~4 and (**b**) D5~7 from the Central Weather Bureau of Taiwan and the Joint Typhoon Warning Center (JTWC). The TC center is indicated at every 6 h. This map is generated by the NCAR Command Language (Version 6.4.0) [Software]. (2017). Boulder, Colorado: UCAR/NCAR/CISL/TDD. 10.5065/D6WD3XH5.

**Figure 3:**
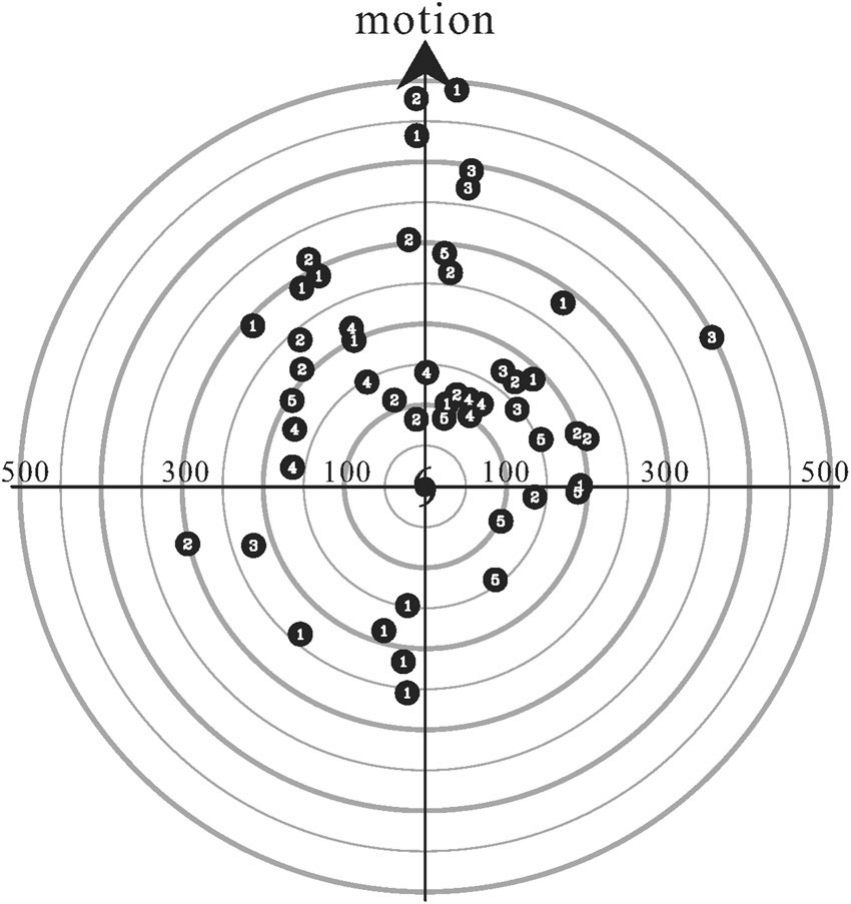
Plan view of all identified TCRs (50 cases) relative to the TC center and motion. The TC motion is towards the top of the page. Each solid dot represents a TCR. Airflow patterns (APs, 1~5) corresponding to each TCR are indicated by white digits. The range rings are indicated every 50 km.

With comprehensive examination of the available radar measurements from 2003 to 2015, a total of 50 TCRs satisfying the above criteria are identified from 22 TC events. Table 2 summarizes the basic information of these identified TCRs. The number of identified TCRs within each of the synthesis domains is also indicated in Fig. 1. For the TCRs passing over the synthesis domains D1~4, located east or north of Taiwan, the corresponding TCs usually tracks northwestward (Fig. 2a). This is simply because the outer rainbands associated with these TCs has greater chances of moving into the synthesis domains from the open ocean before making landfall on the eastern coast of Taiwan. Several of the TCs corresponding to those TCRs that are captured by the synthesis domains D5~7 has a northward track from the Bashi Channel to the Taiwan Strait (Fig. 2b). A plan view of the locations of all analyzed TCRs including their respective TC centers and the direction of TC motion is illustrated in Fig. 3. The outer TCRs exhibits radial distances ranging from ~100 to ~500 km, with the majority of the rainbands confined to the front quadrants. Fewer rainbands are seen in the rear quadrants due to the relative configurations between the generally westward-moving TCs in the northwestern Pacific Ocean and the Taiwan area (cf. Fig. 2). However, the movement of TCs and the vertical shear of the large-scale environment would also be the possible factors contributing to this asymmetry^8^. It should be noted that three TC events [Dujuan (2003), Sepat (2007), and Usagi (2013)] exhibit concentric eyewall structure during the period of dual-Doppler analyses. There are five TCR cases (TCR1, TCR21, TCR22, TCR23, and TCR43) associated with these TCs. Based on the detailed examination of radar observations, these five TCR cases are all located in the outer region well beyond the position of outer eyewall.

**Table 2:** Basic information of 50 TCRs identified in this study.

TCR case	Airflow pattern	Typhoon	Intensity	Synthesis domain	Radial distance (km)	Propagating speed(m s^−1^)	Propagating direction(°)	Cross-band velocity (m s^−1^)	Along-band velocity (m s^−1^)
**1**	3	Dujuan (2003)	Moderate	D4	171.2	35.7	231.5	19.9	29.7
2	1	Mindulle (2004)	Weak	D3	105.1	14.1	316.6	12.7	6.0
**3**	1	Aere (2004)	Moderate	D2	288.0	26.3	190.7	7.4	25.2
4	1	Aere (2004)	Moderate	D2	290.8	25.8	198.5	18.2	18.3
**5**	2	Nock-Ten (2004)	Moderate	D2	83.1	25.2	285.8	18.1	17.6
**6**	2	Nock-Ten (2004)	Moderate	D1	112.8	24.5	279.7	20.0	14.1
**7**	2	Haitang (2005)	Strong	D2	478.2	21.1	228.2	17.4	11.9
**8**	1	Haitang (2005)	Strong	D2	432.4	19.9	235.5	9.7	17.4
**9**	1	Haitang (2005)	Strong	D1	490.2	23.7	240.5	12.8	20.0
**10**	2	Matsa (2005)	Moderate	D2	237.5	27.7	176.8	5.3	27.2
**11**	2	Matsa (2005)	Moderate	D1	314.3	22.5	207.9	13.8	17.8
12	2	Matsa (2005)	Moderate	D2	209.5	10.0	239.9	10.0	0.6
13	4	Matsa (2005)	Moderate	D2	175.8	26.2	155.6	4.1	25.9
14	3	Talim (2005)	Strong	D2	393.2	23.0	210.4	12.8	19.1
15	2	Talim (2005)	Strong	D2	119.2	13.8	246.3	7.8	11.4
**16**	2	Talim (2005)	Strong	D1	198.1	33.9	275.1	14.4	30.7
17	3	Damrey (2005)	Weak	D3	398.1	16.7	298.9	2.9	16.4
**18**	2	Longwong (2005)	Strong	D2	169.6	28.4	241.1	1.9	28.3
**19**	2	Longwang (2005)	Strong	D1	207.8	29.0	275.4	15.9	24.2
**20**	1	Longwang (2005)	Strong	D1	191.5	26.6	286.6	7.6	25.5
21	3	Sepat (2007)	Strong	D3	371.6	20.2	245.2	10.6	17.2
22	5	Sepat (2007)	Strong	D3	288.1	21.5	251.6	11.2	18.3
23	5	Sepat (2007)	Strong	D3	86.9	16.5	295.9	13.1	10.0
24	5	Krosa (2007)	Strong	D3	195.4	16.4	252.8	14.9	6.9
**25**	4	Krosa (2007)	Strong	D2	147.1	11.5	235.4	4.7	10.5
**26**	4	Krosa (2007)	Strong	D1	119.4	16.7	251.9	6.2	15.5
**27**	4	Krosa (2007)	Strong	D1	102.8	13.1	287.5	9.0	9.5
**28**	4	Sinlaku (2008)	Moderate	D1	140.3	24.4	225.7	3.8	24.1
**29**	4	Sinlaku (2008)	Moderate	D1	122.5	23.2	225.7	10.1	20.9
**30**	4	Jangmi (2008)	Strong	D4	215.1	29.6	195.8	14.5	25.8
**31**	2	Jangmi (2008)	Strong	D2	304.8	24.1	246.3	6.0	23.3
**32**	4	Jangmi (2008)	Strong	D4	165.5	16.5	134.1	7.5	14.7
**33**	2	Jangmi (2008)	Strong	D1	265.2	28.0	260.2	9.7	26.3
**34**	1	Molave (2009)	Weak	D4	187.2	23.0	279.7	10.1	20.6
35	3	Meranti (2010)	Weak	D7	147.7	19.4	329.9	18.7	5.2
36	5	Meranti (2010)	Weak	D7	102.5	24.6	6.2	0.7	24.6
**37**	2	Talim (2012)	Weak	D6	135.6	18.8	79.4	8.3	16.9
38	1	Saola (2012)	Moderate	D2	200.2	23.8	239.0	8.3	22.3
39	1	Saola (2012)	Weak	D8	238.7	11.5	45.8	3.4	11.0
40	3	Soulik (2013)	Weak	D7	223.9	21.2	49.5	1.7	21.1
41	1	Trami (2013)	Weak	D7	290.5	16.1	173.6	13.1	9.3
**42**	2	Trami (2013)	Weak	D6	301.6	15.6	66.0	0.5	15.6
43	5	Usagi (2013)	Moderate	D6	188.1	33.9	301.6	2.6	33.8
44	1	Matmo (2014)	Moderate	D2	282.6	19.5	278.2	8.6	17.5
**45**	1	Matmo (2014)	Moderate	D7	148.5	17.9	50.0	2.4	17.7
46	5	Matmo (2014)	Moderate	D8	143.8	20.5	39.2	12.2	16.5
**47**	1	Matmo (2014)	Weak	D7	185.0	19.9	48.4	6.8	18.7
**48**	1	Matmo (2014)	Weak	D7	217.7	18.3	54.9	3.8	17.9
49	1	Matmo (2014)	Weak	D7	255.6	23.6	35.7	−5.9	22.8
50	5	Linfa (2015)	Weak	D6	153.8	25.2	312.3	14.9	20.3
Mean					219.9	21.8		9.3	18.4
Std dev					100.3	5.9		5.8	7.0

Bolded numbers in the first column (TCR case) represent those TCR cases with available surface observations.

Previous studies of TCRs have suggested that convective characteristics would be probably different over different stages and different segments of an outer rainband due to the evolution of moist convection^8,74^. For the present study, the analyzed outer TCRs are all initiated well offshore beyond the observational range of the ground-based Doppler radars (Fig. 1) so our dual-Doppler analyses cannot capture the rainbands in their formative stage. A consequence of radar images from each of the studied TCRs as they move into the observational coverage by coastal radars are examined to provide information of what stage and which sector of the studied rainbands. The investigation indicates that the precipitation intensities for most of the rainbands (68%) remain similar whereas relatively fewer rainbands (28%) are observed to intensify. The smallest percentage of the rainbands (4%) is in the weakening stage. Therefore, the convective structures presented in this study are most relevant to mature or developing TCRs instead of their formative or decaying stages. In addition, roughly 60% (40%) of the studied TCRs are captured by the dual-Doppler analysis in their downwind (upwind) segment. It is also found that although ~25% of the studied TCRs tend to have more convective (stratiform) precipitation features in the upwind (downwind) segment, there are no obvious differences in convective characteristics (i.e., convective vs. stratiform) between the upwind and downwind sides for most of the studied TCRs (~70%). This implies that the results from the present study would not influenced significantly by the along-band variations in the convective nature.

The movement of each of the studied TCRs for the given period of synthesis is obtained by tracking the leading edge of their 35 dBZ contour using a sequence of low-level PPI scans of radar reflectivity. The propagating speeds of the studied rainbands are observed to vary case by case (~10–30 m s^−1^), with very few cases above 30 m s^−1^. The rainbands are embedded within the large-scale cyclonic TC circulation so they exhibit an obvious propagating motion not only in the cross-band direction but also in the along-band direction (Table 2). In the present study, the TC centers are frequently located well offshore in a longer distance to radars, as the studied outer TCRs pass over coastal synthesis domains (cf. Fig. 1). Making an accurate estimate for the rainband’s motion relative to the TC motion is usually not practical because of difficulties in determining TC center locations from radar observations. Along-band variations in the radial distance due to the spiral nature of the outer TCRs represent another complexity to this velocity estimate. Despite these uncertainties, a crude estimate for the relative distance of the studied TCRs to the TC center is possible if both the rainband and TC eye could be visually tracked via radar-observed precipitation patterns over a considerable period of time. It is found that more than a half of the studied rainbands tend to move away from the TC center (i.e., outward propagation) while only a small portion of the rainbands (~18%) are observed to be approximately stationary relative to the TC center. The outward propagating or stationary characteristic is similar to that of previously documented outer TCRs and principal band^5,11,38^.

### Dual-Doppler analysis

In this study, the dual-Doppler synthesis of the multiple-view reflectivity and radial velocity data^75^ is performed to retrieve the three-dimensional precipitation and wind information of the TCRs. Based on the consideration of synthesized geometries, the seven domains located offshore, surrounding the Taiwan island, are determined for dual-Doppler synthesis, as indicated by the inset boxes (D1~D7) in Fig. 1. The cross-beam angles of each pair of synthesized radar within the domains are between 30° and 150° so they could produce relatively smaller uncertainties and errors in the dual-Doppler-derived winds^76^. Distances of radar sites to the studied rainbands are typically between 30 and 90 km so as to provide better sampling of winds at lower and upper levels. If we take the lower bound of radar beams, earth curvature, and the heights of radar sites into account, the average altitude of the lowest PPI scan near the rainband’s location is calculated to be ~0.6 km (MSL), which is comparable to the typical lowest level of available data (0.5~1 km) from previous dual-Doppler analysis works of TCRs and squall lines^54,77,78^.

For the procedure of the dual-Doppler synthesis, the National Center for Atmospheric Research (NCAR) SOLO software^79^ is first used to unfold the radial velocities and to remove incorrect or obviously suspicious values of radar reflectivity and radial velocity. Consequently, the NCAR REORDER software^80^ is used to interpolate reflectivities and radial velocities from the raw PPI scans to Cartesian coordinates with a horizontal grid spacing of 1 km and a vertical grid spacing of 0.5 km, with the lowest (highest) analysis level located at 0.5 (10) km (MSL). Given the horizontal data spacing (~1.5 km) at the range of 90 km and the average vertical data spacing (~1 km), the horizontal (vertical) grid spacing chosen herein is roughly comparable to the horizontal (vertical) data spacing divided by 2.5 (i.e., ∆/2.5)^81^. In the interpolation procedure, the advection adjustment due to the rainband’s movement is also considered^66,82^. The NCAR software program Cartesian Space Editing, Synthesis, and Display of Radar Fields under Interactive Control (CEDRIC^83^) is used for the synthesis of the gridded radial velocities into horizontal wind fields. The vertical air motions are obtained through the variational adjustment of the anelastic continuity equation with boundary conditions of zero vertical motions at the surface and the echo’s top.

**Figure 4:**
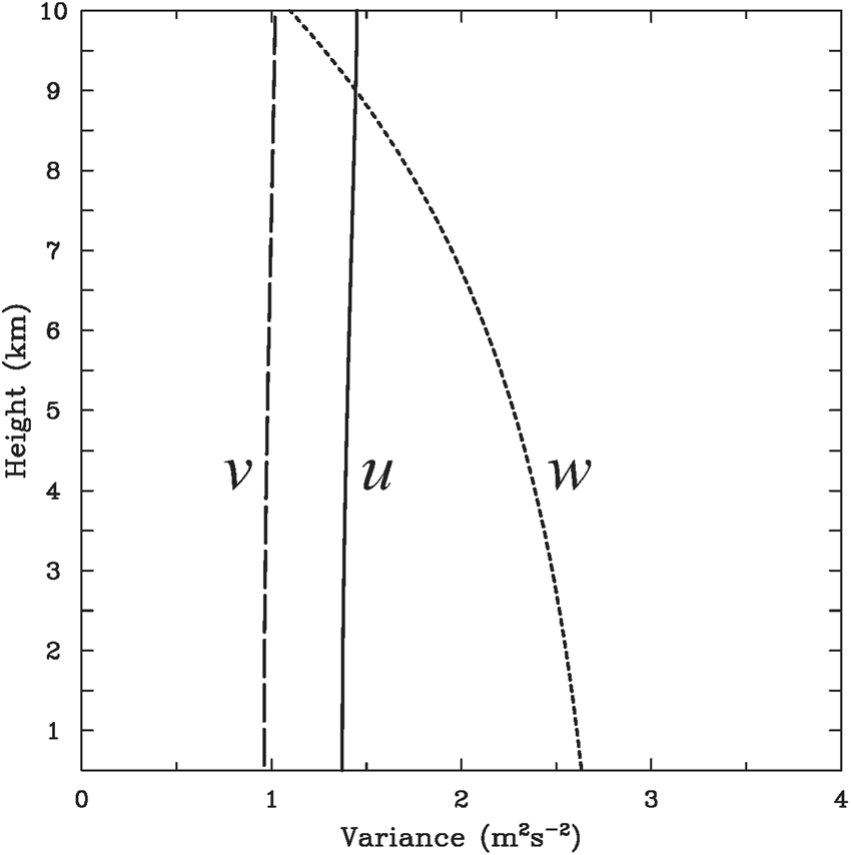
Vertical profiles of the mean variances of *u*, *v*, and *w* errors averaged for all studied rainbands based on the synthesis geometry and grid spacing adopted in this study.

Accuracy of dual-Doppler-derived winds is influenced by various sources of errors due to incomplete sampling, boundary conditions, and integration procedures^76^. Following Ray *et al*.^75^ and Kessinger *et al*.^84^, the error characteristics of synthesized winds may be evaluated for each studied rainband based on synthesis geometry and grid spacing adopted herein. To better elaborate representative errors in regions encompassing the studied TCRs, the mean variances of wind errors averaged for all rainbands are calculated and illustrated as a function of altitudes, as shown in Fig. 4. In this analysis, the variances of radial velocity and terminal velocity are assumed to be 1 m^2^ s^−2^. The boundary condition for the vertical velocity variance (1 m^2^ s^−2^) is applied at the top of the synthesis domain and downward integration is performed. The error variances of horizontal wind components (i.e., *u* and *v*) are small (1~1.5 m^2^ s^−2^) and remain constant with height. The horizontal velocity errors can be considered negligible when compared to strong horizontal winds (typically around 20 m s^−1^) associated with TC circulations. The error variances of vertical velocities (*w*) are slightly larger and range from 1 to 2.7 m^2^ s^−2^. These expected values of *w* errors (1~1.6 m s^−1^) are smaller than the typical strength of updrafts (on the order of 5 m s^−1^ or more) observed within outer TCRs^38,66^.

### Geometric definition

**Figure 5:**
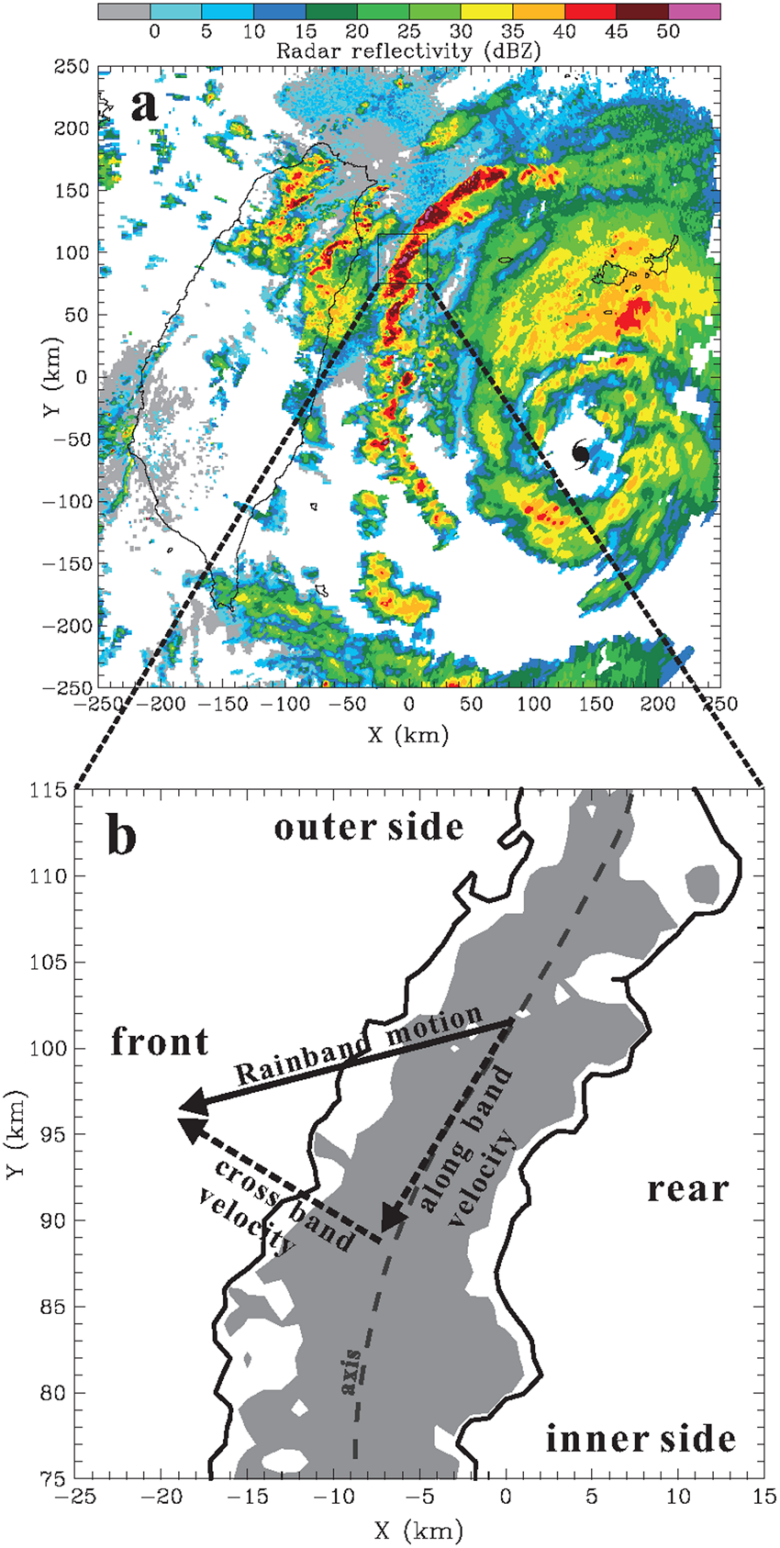
(**a**) Low-level composite radar reflectivity (dBZ) from the CWB radars at 0236 UTC 10 Aug associated with a TCR identified from Typhoon Saola (2012). The inset box shows the zoomed-in area in (**b**). (**b**) The geometric definition of the rear and front side for the TCR. Radar reflectivities greater than 35 and 40 dBZ are indicated by contour and shading, respectively. In this case, the rear (front) side is exactly equivalent to the inner (outer) side as the rainband moves towards the outer side of the typhoon. The definition of the cross-band and along-band velocities are also highlighted, with positive values away from the typhoon center and in the direction of the cyclonic typhoon circulation, respectively. This map is generated by the NCAR Command Language (Version 6.4.0) [Software]. (2017). Boulder, Colorado: UCAR/NCAR/CISL/TDD. http://dx.doi.org/10.5065/D6WD3XH5.

For a convenient depiction of the rainband’s structures, we have adopted the terminology “front” and “rear” in a manner similar to that used for squall lines. A schematic diagram from an outer TCR of Typhoon Saola (2012), as shown in Fig. 5, illustrates the geometric definition of these terms. Because this particular rainband moves roughly toward the southwest, front refers to the western side of the band and rear refers to the eastern side of the band. Moreover, given that the rainband is located northwest of the typhoon center (Fig. 5), the eastern side of the rainband is closer to the center, and thus it is also convenient to refer to it as the inner side. In this case, the rear and front sides are exactly equivalent to the inner and outer sides, respectively, as the rainband moves towards the outer side of the typhoon (i.e., positive cross-band moving velocity shown in Fig. 5). However, if a TCR advances toward the inner side, the rear (front) side of the rainband should refer to outer (inner) side. As indicated in Table 2, the studied TCRs usually moved towards the outer side, except for the one rainbands (case 49) that possess a negative cross-band moving velocity. Therefore, the geometric configuration of Saola’s TCR, shown in Fig. 5, is the dominant condition for the studied rainbands. In our discussions below, we have considered the rear (front) side as the inner (outer) side for convenience of presentation.

### Other data

In addition to the radar measurements and dual-Doppler synthesis as described above, 1-min temporal resolution surface observations available within the coastal area of Taiwan are used to retrieve detailed surface characteristics associated with the studied TCRs. It should be noted that some spatial and temporal variations in the thermodynamics along the different portions of the rainbands are possible^69^. To mitigate this uncertainty, data from a number of particular coastal and island stations (location in Fig. 1) subject to the passage of some of our studied TCRs and also nearest to the synthesis domains are chosen for analysis. More than a half of our studied TCRs (28 cases, bolded in Table 2) are available for this investigation and these surface observations provide valuable thermodynamic information to complement the dual-Doppler-derived kinematics. The National Centers for Environmental Prediction (NCEP) Final Operational Global Analysis data (1° × 1°) are also used to analyze the large-scale environmental conditions associated with the studied TCRs.

## Results

### Structural classification

**Figure 6:**
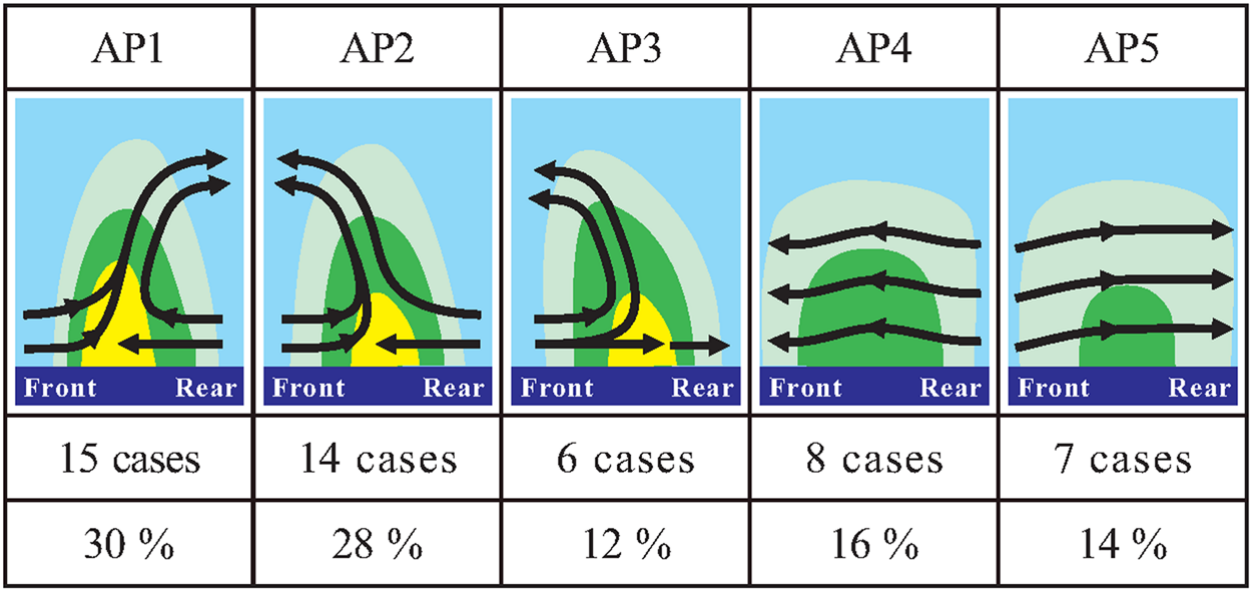
Schematic band-normal vertical cross sections qualitatively illustrating the band-relative kinematic structures and their associated precipitation for five different APs identified from the studied TCRs. Heavy solid arrows indicate salient airflow features observed from the dual-Doppler analyses, and color shading denotes the generalized precipitation pattern associated with the studied TCRs. The number of the TCR cases and frequencies of occurrence for each AP are also indicated.

To understand the common airflow and precipitation structures of TCRs, we undertook a comprehensive examination of the dual-Doppler-synthesized fields for all of the studied TCRs. In reality, some temporal and spatial variations in the details of the rainband structure, presumably due to convective-scale motions, are expected. These rapidly evolving smaller-scale variabilities are inherently complicated but are not the focus of the present study. The primary attempt herein is to seek the rainband-scale, more-robust structural characteristics by looking into a large set of the band-normal vertical cross sections of the airflow and precipitation for all analyzed TCR cases. These analyses enable us to get a sense of the representative airflow and precipitation patterns for each of the different rainbands. With this approach, it was found that the structural characteristics of the outer TCRs could be fundamentally classified into five distinct band-relative airflow patterns (APs). These APs and their associated precipitation features are illustrated in Fig. 6. The specific APs corresponding to each of the studied rainbands are also indicated in the second column of Table 2.

AP1 exhibits a rearward tilting updraft rooted in the prominent low-level convergence between the rear-to-front flow (hereafter RTF) and front-to-rear flow (hereafter FTR). AP2 is similarly characterized by an obvious low-level convergence between the RTF and FTR. However, an overturning updraft (i.e., frontward tilting updraft aloft) is evident. AP3 has an overturning updraft feature but is dominated by the FTR at low levels, which decelerates as it passes through the region of the heaviest precipitation. In contrast to the generally convective nature of the precipitation with prominent vertical extents, horizontal gradients and intense radar reflectivity for AP1~3, the precipitation associated with AP4 (AP5) is less convective, with weaker vertical motions and a deep layer of RTF (FTR) throughout the rainband’s area. Although each of these APs is distributed over diverse regions and without obvious preference in its location relative to the TC center (Fig. 3), most of the rainbands with AP4 and AP5 are located within 200 km of the radial distance. It is likely that the inner-core circulation of TCs may have some impacts on the structural characteristics of these two APs.

### Composite structure

**Figure 7:**
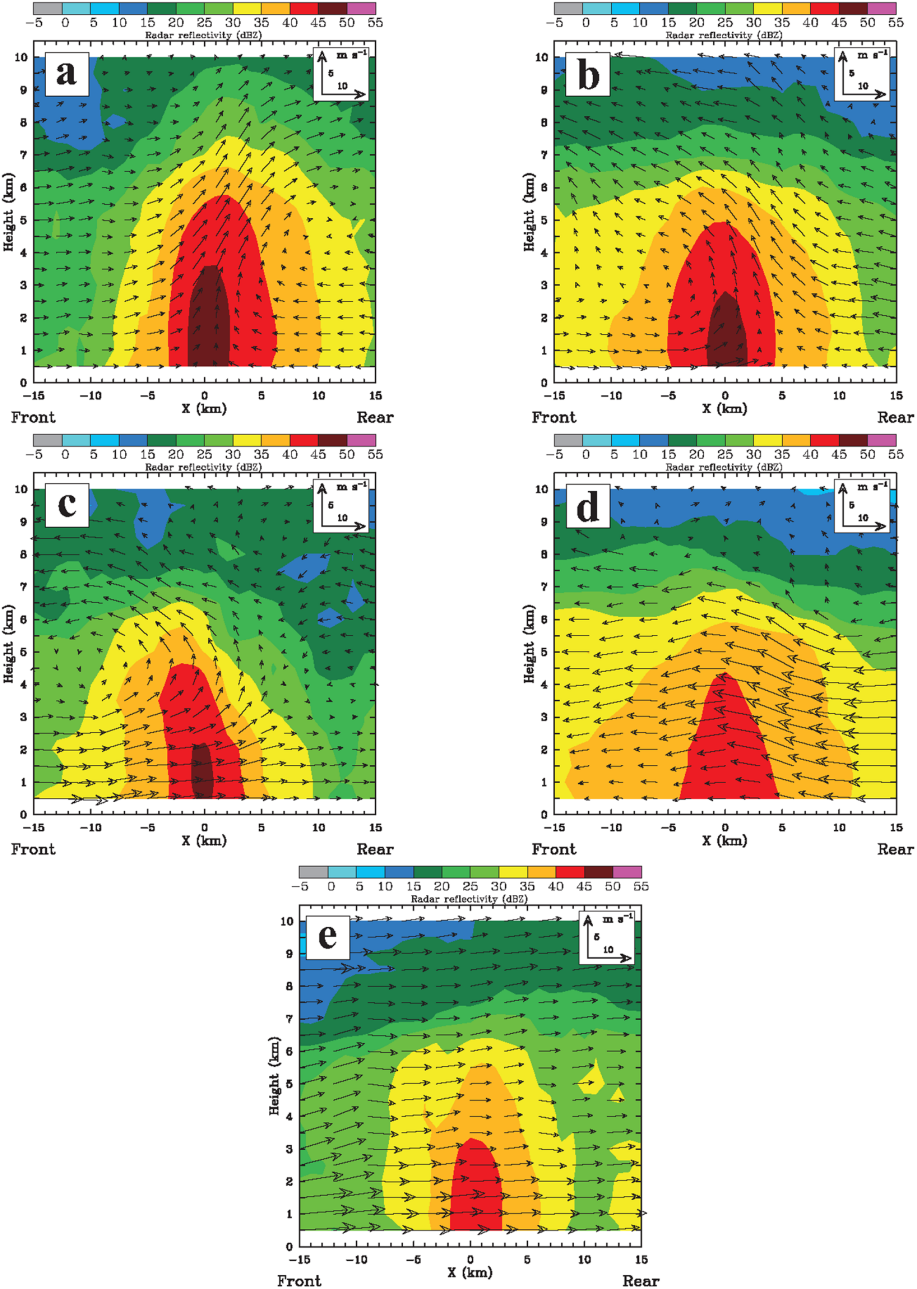
The spatial composite of the band-normal vertical cross sections of the dual-Doppler-derived airflows (indicated by wind vectors) and radar reflectivities (dBZ, color shading) for each AP of the studied TCRs. (**a**) AP1, (**b**) AP2, (**c**) AP3, (**d**) AP4, (**e**) AP5.

To better demonstrate these observed APs, a spatial composite of the band-normal vertical cross sections of the dual-Doppler-derived airflow and precipitation from the different TCR cases for each AP is performed at locations of the heaviest precipitation within the rainband. The composite enables presentation of a reasonable number of figures to illustrate the key aspects of different APs seen from dual-Doppler observations. The composite band-relative structures shown in Fig. 7 reveal important signatures of airflow and precipitation. It should be noted that there are three different methods that can be applied to generate the composite field. The first method is to choose a representative cross section for each of different TCR cases and then to calculate the mean for these sections for a given AP. The second method is simply to take the mean of all individual vertical cross sections from different rainband cases for a given AP. The third method is to obtain an averaged vertical cross section first by averaging individual cross sections for each TCR case, and then we take the mean of these averaged cross sections for a given AP. No matter what method we use for the averaging, we found that the composite results are very similar and consistent. In view of this, only the composite results using the first method (i.e., Fig. 7) are presented.

The FTR observed for AP1 tends to meet with the RTF near the outer edge of the rainband to form an obvious convergence zone, convective precipitation, and a rearward tilting updraft (Fig. 7a). The mean magnitudes of maximum convergence below 3 km (MSL) averaged along the rainbands range from 1 × 10^−3^ to 3 × 10^−3^ s^−1^. The FTR is present throughout the troposphere ahead of the band; however, the layer of the RTF is relatively shallow and confined to below ~3.5 km (MSL) on the inner side. These kinematic patterns are highly similar to those of the convective regions of mature squall lines documented previously with dual-Doppler observations^44,77,85–87^.

For AP2, the FTR on the outer side is shallower [below ~2 km (MSL)], and it meets with the RTF to lift the air upward and to form an overturning-updraft-like feature (Fig. 7b). The intensities of low-level convergence are generally comparable to those of AP1. Although the frontward slant of the vertical motions, forced by the flow collision between the RTF and FTR, as revealed in Fig. 7b, is relatively less common for a mature squall line, it has been frequently documented within linear convective systems and/or during the early development of squall lines due to the presence of stronger ambient vertical shear, which is favorable for convective updrafts to lean downshear^47,88,89^.

**Figure 8:**
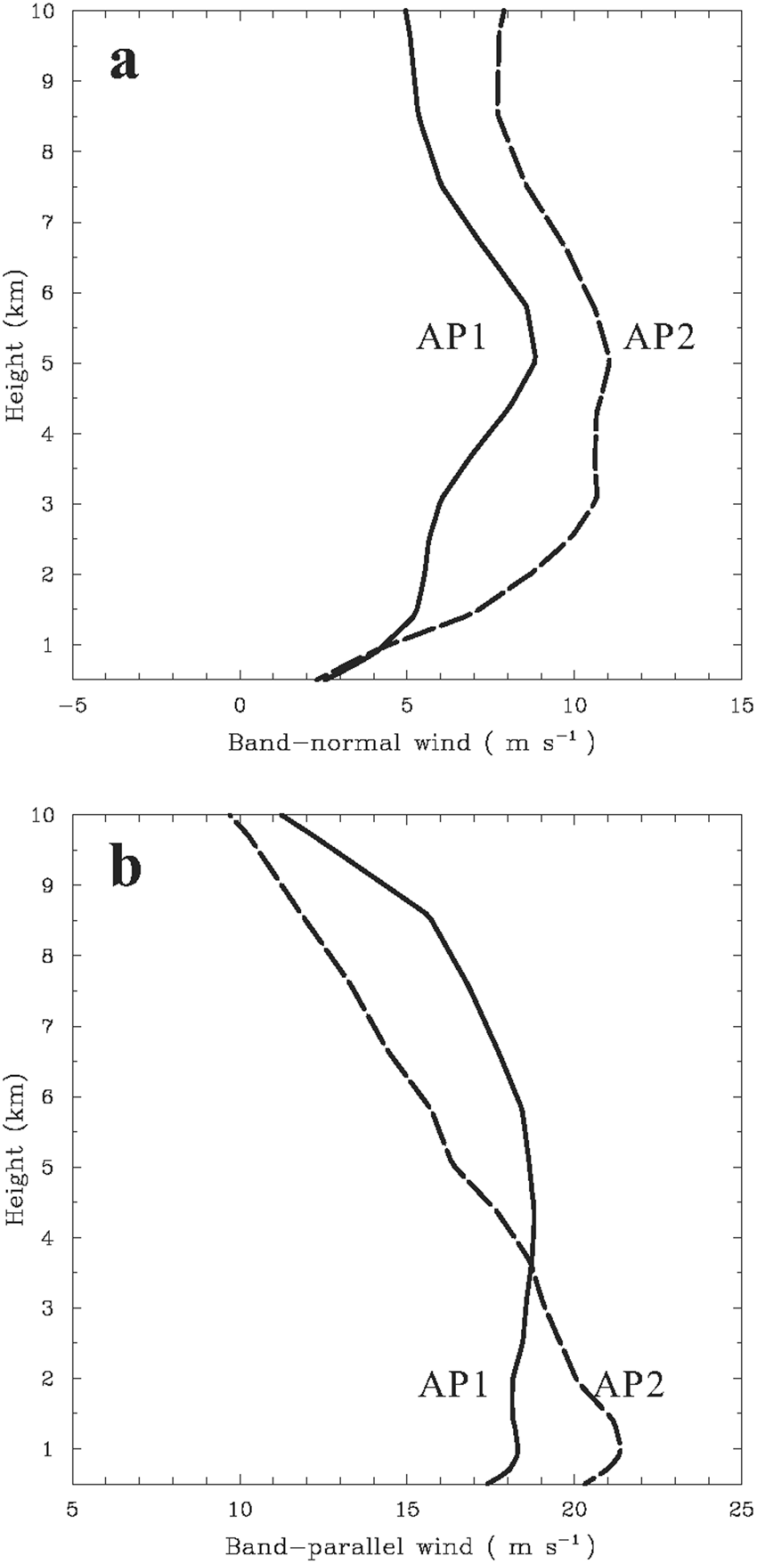
The mean vertical profiles of environmental winds from the NCEP analysis gridded data calculated over the regions ahead of the rainband for the TCR cases with AP1 (solid line) and AP2 (dashed line). (**a**) Band-normal winds, with positive values toward the front (outer) side of the rainbands (**b**) Band-parallel winds, with positive values in the direction of the cyclonic circulation.

Consistent with the scenario above, the mean vertical profiles of the band-normal winds from the selected NCEP analysis gridded data that were located over the regions ahead of the rainbands (i.e., the front side) indicate more pronounced frontward ambient shears (i.e., band-normal winds increase with height) in the lower troposphere for AP2 (Fig. 8a). The mean cross-band vertical wind shear calculated below 3 and 5 km (MSL) is equal to ~3.4 and ~2.0 m s^−1^ km^−1^, respectively. The shear magnitude is comparable to that of tropical squall lines^90^. The rainband’s environment of AP1 is characterized by relatively gentle vertical variations of the band-normal winds (i.e., weaker vertical shear, ~1.4 m s^−1^ km^−1^) at low levels. Despite the potential influence of convectively generated cold pools, the environmental flow with larger shear vectors toward the front side for AP2 is consistent with its overall downshear tilt of updrafts (cf. Fig. 7b). Note that AP2 exhibits stronger band-normal winds (~8–11 m s^−1^) in the mid-to-upper troposphere (Fig. 8a), which would favor occurrence of the upper-level rear-to-front flow (i.e., overturning updrafts aloft) as seen in Fig. 7b. The mean vertical cross sections of radial winds associated with TCs constructed from the NCEP analysis data (not shown) reveal weaker (stronger) intensities of upper-level cyclone-scale outflow for AP1 (AP2), consistent with their relative strength of environmental band-normal winds shown in Fig. 8a. It is thus reasonable to suspect that the upper-level TC outflow characteristics could also be a factor contributing to the observed difference in the rainband’s structures between AP1 and AP2.

In the cases of AP1 and AP2, the intensities of the band-parallel winds are obviously stronger than those of the band-normal winds and decrease with height in the mid-to-upper troposphere (Fig. 8b), both of which reflect typical kinematic features of TC circulations. However, the along-band vertical shear is quite small in the lower troposphere. Note that much weaker along-band vertical shear than the cross-band vertical shear evident at low levels is also the characteristic of the environmental conditions associated with tropical and midlatitude squall lines^90–92^. It is noteworthy that the mean vertical profiles of environmental thermodynamics associated with AP1 and AP2 calculated from the NCEP data (not shown) reveal similar characteristics, with relative humidity of ~85–94% and obvious convective instability [i.e., decrease in equivalent potential temperature (*θ*_*e*_) with height] in the lower troposphere. The differences in *θ*_*e*_ between AP1 and AP2 are minor (<3 K) at the same levels and the minimum in *θ*_*e*_ occurs at ~4–5 km (MSL) for both APs. These two APs account for the majority of our analyzed TCRs (29 cases; ~58%) (cf. Fig. 6). Consistent with this statistic, previous observational studies have shown that the outer TCRs tend to exhibit kinematic structures similar to either AP1 or AP2^66,82^.

Figure 7c–e basically reflect what we have already described for AP3–5, shown in the schematic diagram of Fig. 6. The less intense precipitation of AP4 and AP5, is associated with a broader zone of weaker upward motions (<~2 m s^−1^). Convergence associated with the deceleration of either FTR or RTF at low levels is also evident, with magnitudes of 0.5 × 10^−3^~1.5 × 10^−3^ s^−1^ that are generally weaker than those of AP1 and AP2. Bright-band signatures near the 0 °C level (i.e., between 5 and 6 km MSL) in the radar reflectivity are sometimes present as seen from individual vertical cross sections of these rainbands (not shown). The causal relationship between the low-level convergence and the rainband’s precipitation is somewhat uncertain in this analysis because the stratiform precipitation in the outer region of the TCs would probably also result from the melting process of the upper-level ice particles within the inner-core convection^50^. In this context, the movement and formation of AP4 and AP5 would be influenced by the characteristics of storm-scale radial and tangential flow of TCs.

Each of these three APs (AP3–5) accounts for only 12~16% of our analyzed TCRs (cf. Fig. 6), implying that these APs would not prevail for the outer TCRs. It should be noted that AP3 (cf. Fig. 7c) resembles the common airflow pattern of the previously documented TCRs located closer to the inner core of TCs, such as the principal bands^11,51,54^. Band-normal circulations of the principal band usually exhibit no obvious RTF and are dominated by the low-level inflow from the outer to inner side, with a prominent feature of an overturning updraft aloft. To some extent, AP3 reflects a typical secondary circulation associated with TCs with storm inflow at low levels and an outflow layer in the mid-to-upper troposphere.

It should be noted that examination of individual vertical cross sections of the studied TCRs indicates evidence of convective downdrafts that typically have higher spatial and temporal variabilities. The averaging procedure in the composite analysis as shown in Fig. 7 somewhat smoothes out signatures of downward motions but retains more robust and persistent features of convective motions associated with the rainbands. Mean intensities of downdrafts for AP1-2 are observed to be stronger and have a wider range from −0.5 to −2.5 m s^−1^, in contrast to generally weaker downward motions (<−1.5 m s^−1^) for AP3-5. It is thus likely that if convectively generated downdrafts can feed into low-level flow, they may have a potential role in favoring the intensification of RTF observed in AP1-2.

### Cold pool characteristics

**Figure 9:**
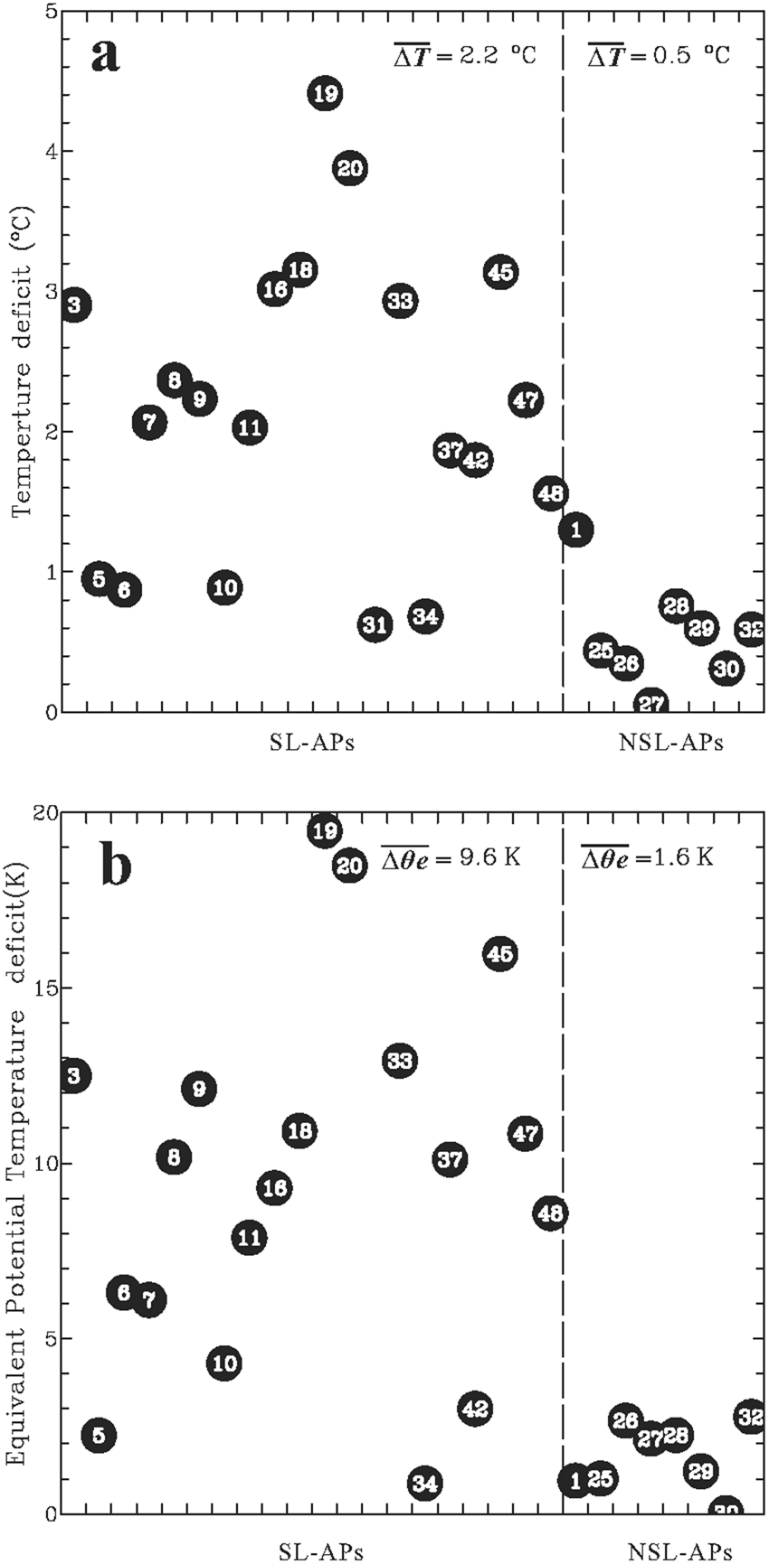
(**a**) Temperature deficits (°C) calculated from the surface observations for the squall-line-like APs (SL-APs, 20 TCR cases) and non-squall-line APs (NSL-APs, 8 TCR cases). Each solid black circle with a white digit (case number in Table 2) represents a TCR case. (**b**) Same as in (**a**), but showing the equivalent potential temperature deficit (*θ*_*e*_, K). The mean deficits for the SL-APs and NSL-APs are also calculated and indicated in (**a** and **b**). Note that the *θ*_*e*_ for the case 31 did not appear in (**b**) because of unavailability of moisture data for this particular TCR.

According to the kinematic structures discussed above, one intriguing question is whether the outer TCRs, with squall-line-like APs (i.e., AP1 and AP2), exhibit obvious cold pools, in a manner similar to squall lines^45,47,48^. The surface observations during the passage of these TCRs are analyzed to provide important clues about this likelihood. Practically, the strength of the convectively generated cold pool may be approximated by the temperature deficit that is defined as the difference between the minimum value found within the rainband and the mean value measured immediately before the arrival of the band (i.e., the pre-rainband environment)^93–95^. As described in the earlier section, 28 TCR cases are available for thermodynamic analysis, and the calculated temperature deficits for each of the squall-line-like APs (20 cases) and other APs (8 cases) are shown in Fig. 9a. The equivalent potential temperature (*θ*_*e*_) deficits are also calculated and shown in Fig. 9b for reference.

The temperature deficits for the squall-line-like APs are characterized by a wider range of values, mostly between 1 to 5 °C (Fig. 9a). More than a half of these APs have prominent temperature deficits greater than 2 °C. These observed temperature deficits are comparable to the intensities of the cold pools reported in the previous studies of the outer TCRs (~2–4 °C)^66,69^ and tropical precipitation^96^. Relatively smaller temperature deficits (around or less than ~1 °C) are also observed in a few squall-line-like APs, such as the TCR cases 5, 6, 10, 31, and 34 (Fig. 9a). Most of these outer TCRs possess frontward tilting updrafts (i.e., AP2; Table 2) with cellular transient and/or stratiform precipitation present ahead of the rainband, as revealed by the radar-observed precipitation distributions (not shown). The near-surface temperature may be modified by the pre-rainband precipitation, which complicates the representation of the calculated temperature deficits as the strength of the cold pools for these particular cases. The temperature deficits for nearly all of the non-squall-line APs are quite minor and generally less than 1 °C. The mean temperature deficit for the squall-line-like and non-squall-line APs are equal to 2.2 °C and 0.5 °C, respectively.

A separation of the calculated *θ*_*e*_ deficits of the squall-line-like APs from those of the non-squall-line APs is also evident (cf. Fig. 9b). A dominant portion of the *θ*_*e*_ deficits for the squall-line-like APs could reach 5–20 K, in contrast to much lower *θ*_*e*_ deficits (<4 K) associated with the non-squall-line APs. The insignificant *θ*_*e*_ deficit for non-squall-line APs would be partly related to weaker downward transport of lower-*θ*_*e*_ air aloft due to weaker convective downdrafts observed within these rainbands as described earlier^97^. Consistent with stronger cold pools observed in the squall-line-like APs, more pronounced changes in kinematics during the passage of these rainbands are also evident, with the average wind shift and deceleration in cross-band flow equal to ~40° and ~8 m s^−1^, respectively. Combination of these surface analyses and dual-Doppler observations presented earlier supports a fundamental similarity between the squall-line-like APs and squall lines.

## Summary and Implications

By performing the dual-Doppler radar analyses from a large set of 50 outer tropical cyclone rainbands (TCRs) associated with 22 tropical cyclones (TCs) that approached the Taiwan area, this study attempts to understand the degree of the prevalence of similarities between the outer TCRs and squall lines. Five distinct airflow patterns (APs) are identified from the studied TCRs, including squall-line-like APs and non-squall-line APs. The outer TCRs with squall-line-like APs (AP1-2) are found to be common (~58%) and are generally characterized by convective precipitation and an obvious convergence zone between the band-relative rear-to-front flow and front-to-rear flow at low levels, with either frontward or rearward tilting updrafts. In contrast, the majority of the non-squall-line APs (i.e., AP4-5) is characterized by less convective precipitation and exhibits a deep layer of either front-to-rear flow or rear-to-front flow within the rainband. Around 12% of the studied TCRs (i.e., AP3) resembles the kinematic characteristics of the previously documented inner TCRs and/or principal bands. Analysis of the surface observations during the passage of the studied TCRs further indicates stronger intensities of the convectively generated cold pools associated with the squall-line-like APs. Combination of dual-Doppler and surface observations supports a fundamental similarity between the squall-line-like APs and squall lines.

This study documents a frequent similarity between outer TCRs and squall lines, which provides important implications. First of all, the effect of cold pools on the initiation and development of moist convection can be expected to be generally prominent in the outer region of TCs, consistent with the previous findings from limited case studies of outer TCRs^66,69^. It has been long recognized that atmospheric cold pools bear high resemblance to the laboratory density current, in terms of their associated airflow patterns and propagation speed^95,98,99^. Nevertheless, the density-current dynamics is commonly treated as a two-dimensional problem, namely, considering the plane of the vertical and the propagation direction. Our knowledge for the behavior of cold pools under strong ambient winds and highly rotational background like the vortex environment of TCs remains very poor. To advance a general understanding of convective forcings for TCRs, future investigations should pay more attention to the dynamics of cold pools and their origin in the TC environment.

In addition, the appearance of TCRs has been traditionally considered as a manifestation of atmospheric waves such as vortex Rossby waves and inertia-gravity waves^27–29,32,37,58^. In particular, these waves usually initiate in the vicinity of the inner-core region and propagate outward radially. Given these wave properties, together with the findings from the present study, one can reasonably anticipate that a dynamic transformation from wave-dominant convection to squall-line-like rainbands is very likely to occur in certain time periods as TCRs propagate outward from inner to outer regions of TCs. As evident in this study, the variability in structural characteristics of the studied TCRs, including both squall-line-like APs and non-squall-line APs, would reflect different stages or different scenarios of such transformation. Carefully tracking the history of evolving and structural details of a given TCR is quite challenging, especially from the observational perspective, but represents a critical, future task to explore the likelihood and understanding of these transformation processes.

## Data Availability

The datasets generated during and/or analysed during the current study are available from the corresponding author on reasonable request.
