# Publisher Correction: The degree of prevalence of similarity between outer tropical cyclone rainbands and squall lines

**DOI:** 10.1038/s41598-019-44434-6

**Published:** 2019-06-06

**Authors:** Cheng-Ku Yu, Che-Yu Lin, Lin-Wen Cheng, Jhang-Shuo Luo, Chun-Chieh Wu, Ying Chen

**Affiliations:** Department of Atmospheric Sciences, National Taiwan University, Taipei, Taiwan

Correction to: *Scientific Reports* 10.1038/s41598-018-26553-8, published online 29 May 2018

The original HTML version of this Article omitted an affiliation for Chun-Chieh Wu and Ying Chen. The correct affiliation is listed below:

Department of Atmospheric Sciences, National Taiwan University, Taipei, Taiwan

This has now been corrected in the HTML version of this Article; the PDF was correct at the time of publication.

